# Has there been a change in the rates of UK sickness certification for back pain over time? An examination of historical data from 2000 to 2010

**DOI:** 10.1136/bmjopen-2015-009634

**Published:** 2016-04-25

**Authors:** Gwenllian Wynne-Jones, Kate M Dunn

**Affiliations:** Arthritis Research UK Primary Care Centre, Research Institute for Primary Care & Health Sciences, Keele University, Keele, UK

**Keywords:** Rate, Absence, EPIDEMIOLOGY

## Abstract

**Objectives:**

This paper aims to investigate historical patterns of sickness certification for back pain from 2000 to 2010.

**Design:**

Electronic medical records from 14 practices that are part of the National Institute for Health Research (NIHR) Clinical Research Network: West Midlands were reviewed. All records for back pain consultations from 2000 to 2010 were downloaded and matched, by date, to corresponding sickness certification records.

**Setting:**

Primary Care.

**Results:**

A total of 93 896 back pain consultations were recorded over the 11-year period, resulting in 30 913 sickness certificates. There was a statistically significant decrease in the rate of certification over the period, falling from 376.8 (95% CI 362.1 to 392) per 1000 back pain consultations in 2000 to 246.5 (95% CI 236.5 to 332.9) per 1000 back pain consultations in 2010. There was also a statistically significant difference in certification between males and females, with males issued more certificates than females. There was a statistically significant difference in certification by age, with those aged 60 years and over being less likely to be issued a certificate compared to all other age groups.

**Conclusions:**

Rates of sickness certification for back pain demonstrated a downward trend between 2000 and 2010. While the reasons for this are not transparent, it may be related to changing beliefs around working with back pain.

**Strengths and limitations of this study**

- This medical record review provides the ability to examine the rate of back pain associated sickness certificates over an 11-year period in all adults registered at 14 general practices.
- The database used has previously been demonstrated to be generalisable to the age and gender of the UK population.
- The reasons for the decrease in sickness certification are not fully clear but may be related to changing beliefs around working with back pain.

## Introduction

Many patients visit a general practitioner (GP) in primary care with symptoms of low back pain; in the UK, each visit to a GP is termed a consultation. While many patients consult their GP for back pain, 85% will not receive a hard diagnosis,^1^ and this consultation will be recorded on the medical record as a consultation for symptoms of back pain. Since so few people with symptoms of back pain receive a hard diagnosis, this paper will use the phrase back pain to include the majority of patients without a diagnosis, in addition to those who do have a diagnosis associated with their pain.

It has been estimated that 38% of adults are affected by back pain in any 1 year at an estimated cost to the National Health Service of £1 billion per annum.^2^ Musculoskeletal pain, principally back pain, is one of the most common reasons for absence from work.^3^ The costs of illness to employers, particularly back pain, are large; it has been estimated that 31 million days of absence were taken as a result of musculoskeletal pain in 2013.^4^ The Chartered Institute of Personnel and Development^5^ reports the rate of UK sickness absence in terms of a percentage of lost working hours, estimating sickness absence as 3.0%, 3.4% and 3.2% of working hours in 2012, 2011 and 2010, respectively. The Health and Safety Executive^6^ has estimated the total net cost of sick pay to be £14.2 billion during the year 2012–2013 as a result of work-related illness and injuries. When it is considered that musculoskeletal pain, principally back pain, is one of the most common reasons for absence from work, it can be assumed that a large proportion of this sick pay is as a result of back pain.^5^ In the UK, proxy measures are used to estimate rates of sickness certification based on work absence. However, reporting rates of absence as a percentage of lost working hours does not reflect the clinical issues associated with certification in terms of the numbers of individuals absent from the workplace, nor does it represent the number of consultations for certification in general practice.^7^ ^8^

The health service costs and lost capacity in the workplace have made health and work a key target for public policy in the UK.^9^ The Government is actively aiming to reduce the number of employees signed off sick each year with a multiagency government programme, launched in 2005, to address the issues of health, work and well-being.^10^ Furthermore, in 2010, the Fit Note was introduced to replace sickness certificates. The aim of the Fit Note was to change the focus from what the patient cannot do to what they can do in relation to work and to provide the GP with the option to state that patients may be fit for some work with provisions: a phased return to work, altered hours, amended duties and workplace adaptations. However, in order to assess the impact of such initiatives, methods are required to estimate rates of sickness certification over time, including the periods before such initiatives were implemented; a measure of the rate of certification per consultation is one method of achieving this.

The National Institute for Health Research Clinical Research Network: West Midlands holds frozen archive data on sickness certification in its Medical Certificates in Primary Care Archive and consultation data in its Consulters in Primary Care Archive. These databases have been validated for assessment of sickness certification,^11^ and the rates of sickness certification for a range of health conditions have been estimated.^12^ However, it is unknown whether the rate of certification for back pain has changed over time or whether there are any trends over time by age and gender. This paper aims to investigate historical patterns of sickness certification for back pain from 2000 to 2010.

## Methods

All consultation records from 2000 to 2010 for individuals with a back pain Read code were downloaded from the Consultation in Primary Care Archive database (CiPCA). Read codes are a hierarchy of morbidity, symptom and process codes, which become more specific further down the hierarchy. A set of Read codes has been established to identify both back pain diagnoses and symptoms consistently from the medical records; this comprises a total of 589 Read codes plus 20 other terms http://www.keele.ac.uk/mrr/morbiditydefinitions/.^13^ It was these codes that were used to identify patients for this study. The inclusion of both symptom and diagnostic codes ensures that both medically unexplained back pain and back pain that has a clear cause are included, as 85% of patients consulting with symptoms of back pain do not receive a diagnosis;^1^ focusing only on those patients with a diagnosis would exclude the vast majority of individuals for whom sickness absence is common.

Records for working age adults (aged 19–64 years), and for those patients who were registered at the practice for the full year included in the analysis, were eligible. Each consultation record includes the unique individual identification number and practice identification number, plus age, gender, and year of consultation, date of consultation, the Read Code which was used to identify the problem with which the patient consulted and the consultation free text. All sickness certification records between 2000 and 2010 for the same individuals, identified using each patient's unique identification number, were downloaded from the Medical Certificates in Primary Care Archive database (MiPCA). In the UK, a sickness certificate is required from the seventh day of absence. Each sickness certification record includes a unique individual identification number and practice identification number, plus age, gender, year of issue, date of issue and the Read Code used to identify the record of a sickness certificate. Sickness certification records were then matched to back pain consultation records using the date of issue/consultation, the patient's unique identification number, plus age and gender.

### Analysis

Numbers of consultations for back pain each year, and numbers of sickness certificates issued, were calculated using SPSS V.21. The rate of certification was defined as the number of certificates issued for back pain divided by the number of consultations for back pain in each year, presented per 1000 back pain consultations. The crude rate of certification and the rates by age and gender were calculated with 95% CIs. A t test for differences in proportions was calculated for year of certification and gender. An analysis of variance (ANOVA) was calculated to examine differences in rates by age. For all calculations, the significance level was set at p=0.001 and a Bonferroni correction was applied to account for multiple analyses in the ANOVA.

## Results

**Figure 1:**
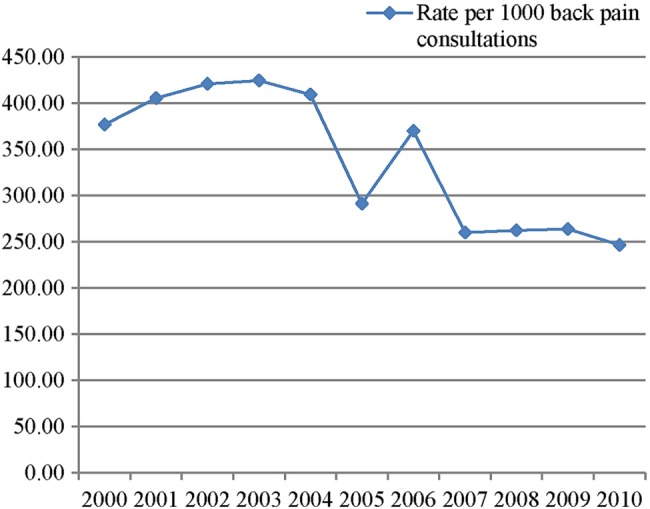
Rates of sickness certification associated with back pain per 1000 back pain consultations.

During the 11-year period (2000–2010), there were a total of 93 896 consultations for back pain, resulting in 30 913 sickness certificates being issued. This gives a sickness certification prevalence of 32.9%, or a third of all consultations for back pain resulting in a sickness certificate. The overall rate of certification was 329.2 certificates per 1000 consultations (95% CI 325.6 to 332.9; table 1 and figure 1).

**Table 1:** Rate of sickness certification per 1000 consultations for back pain by year and gender

				Sickness certification rate per 1000 back pain consultations (95% CI)		
Year	Total number of certificates issued	Total number of consultations	Prevalence (%)	Total	Males	Females
2000	2455	6515	37.7	376.8 (362.1 to 392)	397.1 (375.2 to 419.9)	358.4 (338.5 to 379)
2001	2809	6926	40.5	405.6 (390.7 to 420.9)	484.1 (460.9 to 508.2)	331.5 (312.8 to 350.9)
2002	2863	6801	42.1	420.1 (405.7 to 436.7)	473.7 (450.6 to 497.7)	370.6 (350.7 to 391.4)
2003	3215	7569	42.5	424.8 (410.2 to 439.7)	471.4 (449 to 494.6)	384.1 (365.3 to 403.7)
2004	3060	7476	40.9	409.3 (394.9 to 424.1)	458.2 (436.5 to 480.6)	362.6 (436.5 to 480.6)
2005	2720	9338	29.1	291.3 (280.4 to 302.4)	347.7 (330.3 to 365.8)	242.9 (229.4 to 256.9)
2006	3495	9442	37.0	370.1 (357.9 to 382.6)	394.7 (376.3 to 413.8)	349 (333 to 365.7)
2007	2653	10 200	26.4	260.1 (250.3 to 270.2)	281.8 (266.6 to 297.8)	242.9 (230.2 to 256)
2008	2636	10 046	26.2	262.4 (252.5 to 272.6)	296.1 (280.6 to 312.3)	233.7 (221 to 246.9)
2009	2731	10 349	26.4	263.9 (254.1 to 2739)	293.4 (278.1 to 309.4)	239.5 (226.9 to 252.6)
2010	2276	9234	24.6	246.5 (236.5 to 256.8)	275.7 (259.9 to 292.3)	223.3 (210.6 to 236.6)
All years	30 913	93 896	32.9	329.2 (325.6 to 332.9)	370.5 (364.8 to 376.3)	293.5 (288.8 to 298.3)

There appears to be a downward trend in the rate of certification over the study period, with the rate of certification falling from 376.8 (95% CI 362.1 to 392) per 1000 back pain consultations in 2000, to 246.5 (95% CI 236.5 to 332.9) per 1000 back pain consultations in 2010 (see figure 1). The rate of certification reaches a peak of 424.8 (95% CI 410.2 to 439.7) per 1000 back pain consultations during the year 2003, with another spike in 2006. This decrease in the rate of certification over the 11-year period was statistically significant (p≤0.001).

**Figure 2:**
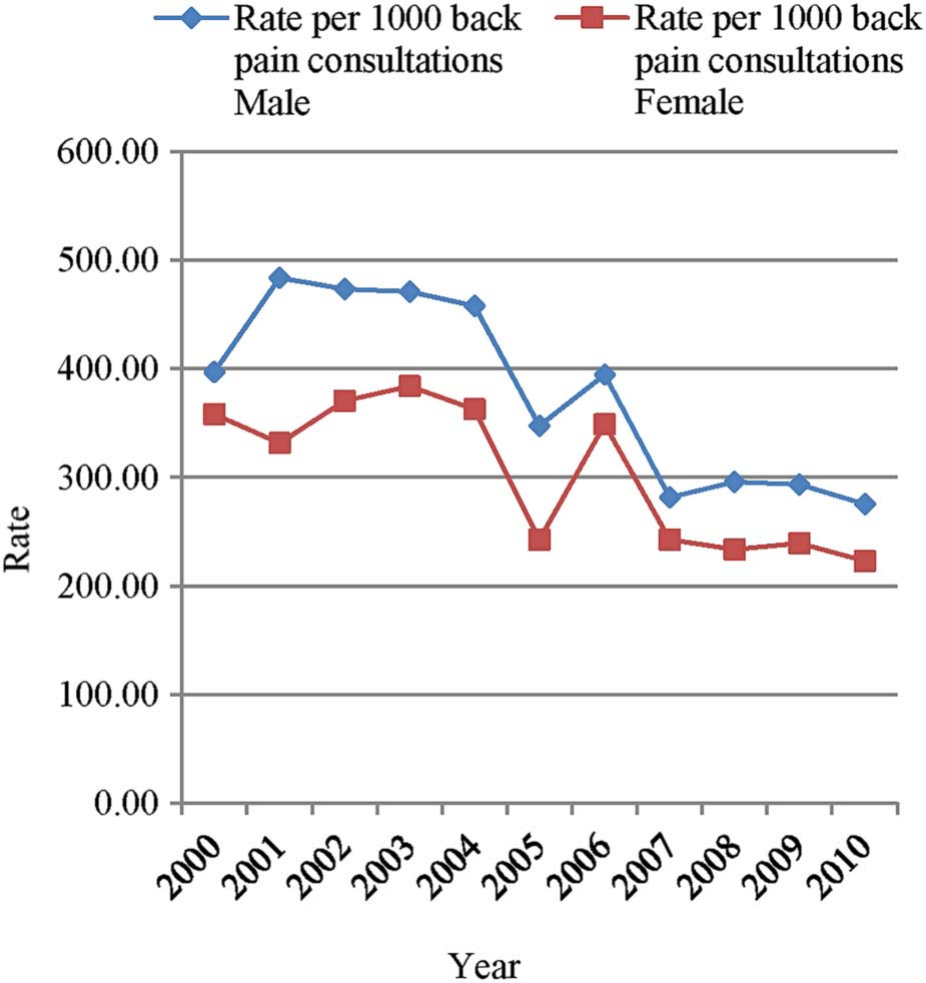
Rates of certification associated with back pain per 1000 back pain consultations by gender.

Examining the data by gender demonstrates that the rate of certification associated with back pain is slightly higher in males compared to females (figure 2). The rate of certification reaches a peak of 484.4 (95% CI 460.9 to 508.2) certificates per 1000 back pain consultations in males in 2001 compared to 384.1 (95% CI 365.2 to 403.7) certificates per 1000 back pain consultations in females in 2003, falling to a low of 275.7 (95% CI 259.9 to 292.3) certificates per 1000 back pain consultations in males during 2010 compared to 223.3 (95% CI 210.6 to 236.6) certificates per 1000 consultations in females during 2010 (figure 2). There was a statistically significant difference in the rate of sickness certification between males and females (p≤0.001).

**Figure 3:**
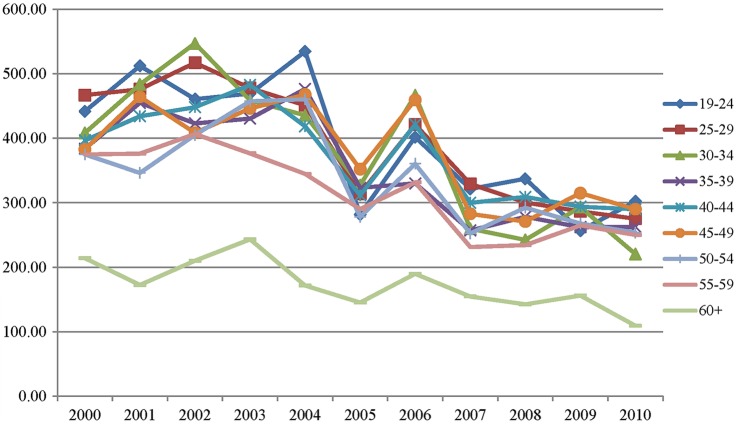
Rates of certification associated with back pain per 1000 back pain consultations by age group.

Comparing the rate of certification per 1000 back pain consultations by age shows that no single age group is reliably recording a higher rate of certification than other groups. However, the 60+ age group consistently record much lower rates of certificates per 1000 back pain consultations compared to the other age groups (figure 3). An ANOVA demonstrates that this difference in rate of certification between the 60+ age group and the other age groups is significant p≤0.001; excluding the 60+ age group from the ANOVA demonstrates that there are no statistical differences in the rate of certification between the age groups from 19 to 59 years.

## Discussion

Overall, the rate of sickness certification for back pain has declined significantly between 2000 and 2010. This decline in rates has principally occurred from 2003 onwards. This study demonstrated that approximately one-third of consultations for back pain will lead to a sickness certificate; this finding is in line with many studies examining back pain which also find that approximately one-third of participants are absent from work.^14^ ^15^

There was a trend in certification by gender, with men reporting consistently higher rates of certification than women. This is to be expected and has been reported in previous studies of certification using this data set.^12^ ^16^ The most likely explanation is the increased proportions of males working in the manual sector at 34.6% when compared to females at 10.9%.^17^ On average, manual workers report more absence than non-manual employees, and also report more absence as a result of musculoskeletal conditions than non-manual employees.^5^ The differences in certification for gender may also be associated with differing consultation behaviours, and it has been reported that men are more likely to consult with an episode of back pain than women at a female:male rate ratio of 1:20 (95% CI 1.13 to 1.28).^13^

There was no clear trend in certification by age with the exception of the 60+ years age group, which consistently had a lower rate of certification compared to younger age groups. Again, the rate of certification in this group showed a downward trend, but it was not as pronounced as in other groups. There are a number of reasons why this difference may be seen in the 60+ age group. First, there are fewer people employed in this age group; in 2011, the Organisation for Economic Co-operation and Development reported that 56% of the population in the 55–64 years age group were employed compared to 80% of the population in the 25–54 years age group.^18^ Second, data from 2013 demonstrated that while individuals in the 50–64 years age bracket are less likely to work on a part-time basis (just 28% of all workers in this age group), those in the over 64 years age group are more likely to report that they work part time, a total of 66% of all those in this age group.^19^ Third, there could be a healthy worker effect whereby individuals suffering with back pain are removing themselves from the workplace. Findings are not likely to be related to differential reporting, as reporting of back pain over time is likely to be consistent.^20^

It is difficult to identify the reason for the decreasing rate of sickness certification in this study. It could be argued that the prevalence of back pain has decreased over time and so there is less need for certification. However, the literature suggests that the prevalence of back pain has remained largely unchanged over the period 1990–2010^21^ and may even have increased.^22^ ^23^ It could also be that the number of consultations for back pain has increased; however, the literature again suggests that this is not the case.^13^ ^24^ ^25^ During the period of analysis that this paper spans, there have been a number of initiatives, in the UK and worldwide, surrounding the management of back pain that may have contributed to the decline in rates of certification. A review of clinical guidelines for the management of non-specific back pain assessed guidelines published between 2000 and 2008;^26^ this review is an update of a previous review.^27^ The authors report that the most common advice is to reassure patients and encourage them to remain active; however, in contrast to the earlier review, the current guidelines increasingly mention return to work, despite back pain, in their recommendations. In the UK, 2009 saw the publication of two National Institute for Clinical Excellence (NICE) reports, the first considering early management of non-specific back pain^28^ and the second reporting on primary care management of long-term sickness absence.^29^ The non-specific back pain guidelines^28^ concur with the review by Koes *et al*^27^ that individuals should be encouraged to remain active and continue normal activities as far as possible. The second report is focused on promoting the benefits of working with health conditions,^29^ specifically back pain, and again encourages individuals to maintain work despite pain. However, the impact of these recent reports is unlikely to be seen in the current analysis.

Recent research from the Global Burden of Disease study has demonstrated that back pain leads to more years lived with disability than any other condition and that this burden of back pain is increasing with an ageing population.^21^ There is also evidence that the number of consultations for back pain has remained static, at least in the population of the USA, for a period of over 10 years from the 1990s to the mid-2000s.^30^ It is interesting then to note that, despite the relative stability of consultations for back pain and the relatively adverse outcomes in terms of the burden of back pain, the number of sickness certificates for back pain has decreased in this study. Within the UK, there have not been any recent public health initiatives which may account for this decrease in certification; there has, however, been a sea change in the information that GPs are advised to give their patients in regard to working with pain. There is consistent information provided by the Royal College of General Practitioners,^31^ Department for Work and Pensions^32^ and NICE,^28^ ^29^ coupled with the availability of information booklets detailing the management of health and work for the GP, patient and employer.^33–36^ It may be that media campaigns to promote working with musculoskeletal pain could see the rate of sickness certification reduce further. There has been some success with media campaigns in Scotland and Australia demonstrating that positive messages around working with musculoskeletal pain led to improvements in knowledge, attitudes and beliefs at the population level which were maintained, albeit at a reduced level, 3 years later.^37–39^ A Canadian study also utilising a media campaign had a more limited effect on behaviours related to back pain, for example, healthcare use, than those run in Scotland and Australia.^40^ However, the Canadian study did find that participants agreement with the statement ‘if you have back pain you should try to stay active’ significantly increased.^40^ It seems then that knowledge does not necessarily translate into behaviour and it has also been reported that provision of information alone is not sufficient to prevent work absence,^41^ but it seems that improving the baseline understanding of the general population may enhance any further information provided by healthcare professionals.^42^

There are a number of limitations to the current study. First, capturing the duration of sickness absence is not possible in the current data set, meaning it is unclear whether the data are unduly influenced by a large number of short-term or long-term certificates. The data presented are a ‘pure’ rate of certification, that is, a rate of certification per consultation. The data do not take into account the potential for a single patient to be issued multiple certificates for one episode of back pain. The possibility that the rate is artificially inflated as a result of a few patients receiving multiple certificates or deflated as a result of multiple patients receiving one certificate of a long duration cannot be ruled out. However, the most frequently recorded duration for absence is 2 weeks,^43–46^ and there is no reason to believe that GPs in the current study would differ to any great degree. It is also important to acknowledge that for approximately two-thirds of people with an episode of back pain, a return to work within 1 month is expected,^15^ indicating that the majority of consultations will be for individual episodes of back pain and therefore be individual certificates.

Second this data set is based in one area of the UK, North Staffordshire, and it could be argued that it is not generalisable to the rest of the population. Previous work with this data set has demonstrated that crude rates of certification change very little when the data are standardised to the age and gender of the population as a whole, and there is no indication that this should be any different for this study.^12^ Lastly, the data included in the manuscript only goes as far as 2010 as from this date onwards the Read coding for sickness certificates changed as a result of the introduction of the Fit Note. This change in Read coding means that Fit Notes are now coded as ‘not fit for work’ and ‘may be fit for some work’. Since this classification is different from the pre-2010 sickness certificates, it would make comparison of the data between these two periods difficult in this manuscript because we would not be comparing like with like. As a result, we could not be confident that any changes in the rate of certification would be as a result of the change to the Fit Note system or as a result of the change in Read coding, that is, more read codes available to classify not fit for work and may be fit for some work.

The main strength of this study is the large database in which this work was carried out. This data set allowed the linking of consultation data to certification data to enable trends by age and gender to be seen. This is the first study to map trends in certification for back pain in the UK using an established data set which has been validated for use in examining both consultation and sickness certification data.^11^ ^13^ ^47^ There is a need for establishing baseline rates of certification against which any change in policy or strategy at either the local or national level can be compared, and this data set goes some way to establishing these baseline figures. Linaker *et al*^48^ state that improvement of existing data sets would be more attractive than the development of a new national system to record sickness absence; with the introduction of the e-Fit Note, the current data set is being updated to include information on duration of absence and whether or not a patient may be fit for work, further strengthening this data set for future research. Another strong point of this study is that is appears to be supported by other literature; this lends credibility to our findings. For example, Ruseckaite *et al*^49^ reported a significant decrease in certification for musculoskeletal disorders between 2003 and 2010 in Australia. They also report a decrease in the rate of certification for back pain and strains, although there is no indication of whether this trend is significant or not. Gabbay *et al*^50^ also reported a decrease in the number of Fit Notes, lasting over 12 weeks, issued over a 12-month period. Lambeek *et al*^51^ reported that the costs of back pain in the Netherlands decreased between 2002 and 2007, acknowledging that a large proportion of this cost is made up of absence. In the UK, the Office for National Statistics has reported a decrease in reported absence across all health conditions between 1993 and 2013.^52^ However, work still needs to be carried out to investigate why there has been this change, and whether it is truly related to the available evidence about working with pain.

## Conclusions

Rates of certification for back pain demonstrated a significant downward trend over the period 2000 to 2010; the reasons for this are not fully transparent but may be as a result of changing beliefs around working with back pain. These findings may provide a benchmark against which the impact of public health initiatives may be evaluated and monitored. With the new recording of the e-Fit Note, this data set will become more useful in tracking rates of certification over time.
